# Impact of COVID-19 on national health screening participation and chronic disease detection: evidence from South Korea’s NHIS database

**DOI:** 10.3389/fpubh.2025.1721010

**Published:** 2026-03-18

**Authors:** Sunyoung Kim, Hyejin Lee, Jungha Park, Sujeong Han, Hye Yeon Koo, Eunbyul Cho, Haeung Song, Bumjo Oh

**Affiliations:** 1 Department of Family Medicine, Kyung Hee University Medical Center, Seoul, Republic of Korea; 2 Department of Family Medicine, Seoul National University Bundang Hospital, Seongnam, Republic of Korea; 3 Department of Family Medicine, Seoul National University College of Medicine, Seoul, Republic of Korea; 4 Department of Family Medicine, SMG-SNU Boramae Medical Center, Seoul, Republic of Korea; 5 Department of Family Medicine, Hallym University Dongtan Sacred Heart Hospital, Hwaseong, Republic of Korea; 6 Department of Cardiology, Gimpo Woori Hospital, Gimpo, Republic of Korea

**Keywords:** COVID-19, chronic disease detection, health system resilience, national health screening program, South Korea, preventive healthcare policy

## Abstract

**Background:**

The COVID-19 pandemic disrupted preventive healthcare services worldwide, raising concerns about missed diagnoses of chronic diseases. South Korea’s national health screening program experienced a substantial decline in participation during 2020. This study investigated how changes in screening rates influenced chronic disease detection and what these shifts reveal about the resilience of the national health system.

**Methods:**

Using the Korea Disease Control and Prevention Agency-National Health Insurance Service (K-COV-N) database, we analyzed trends in national health screening participation (2010–2020) and new diagnoses of hypertension, diabetes mellitus, and dyslipidemia before (2019) and during (2020) the COVID-19 pandemic. Analyses were stratified by sex and 10-year age groups to partially control for confounding factors. Relative risks (RRs) and 95% confidence intervals (CIs) for newly diagnosed chronic diseases were calculated for individuals who underwent health checkups and those who did not.

**Results:**

Health screening participation dropped from 75.4% in 2019 to 68.7% in 2020. Despite this decrease, new diagnoses of chronic diseases increased by approximately 550,000 cases in 2020 compared with 2019. Hypertension, diabetes, and dyslipidemia all showed higher relative risks among individuals who underwent screenings than among those who did not. Cardiovascular and cerebrovascular diseases declined, while heart failure increased slightly.

**Conclusion:**

The decline in preventive health screening did not correspond to a decline in chronic disease detection, suggesting a behavioral and systemic shift toward alternative diagnostic pathways. These findings underscore a resilient yet uneven health system, revealing both adaptive strengths and structural vulnerabilities that maintain continuity of essential services during public health crises.

## 1 Introduction

During the coronavirus disease 2019 (COVID-19) pandemic, some form of lockdown was implemented across almost every country in the world. In South Korea, despite the absence of an official government lockdown, many individuals refrained from going outside because of the fear of contracting SARS-CoV-2. This apprehension was particularly pronounced in relation to visiting hospitals, as individuals expressed concerns about potential contamination by the virus. Access to healthcare services for health promotion and prevention has decreased during the COVID-19 epidemic (1).

In South Korea, national health checkups have been performed since 1980; they have affected health promotion, diagnosis, and treatment of chronic diseases and cancer (1). The target population comprises individuals aged ≥20 years in South Korea (2). According to 2018 data from the Korean National Health Insurance Service (NHIS), 23.5% of those who underwent health checkups were diagnosed with diseases, and 30.4% were suspected of having diseases, emphasizing the importance of comprehensive examinations to identify potential health problems (3).

**Figure 1:**
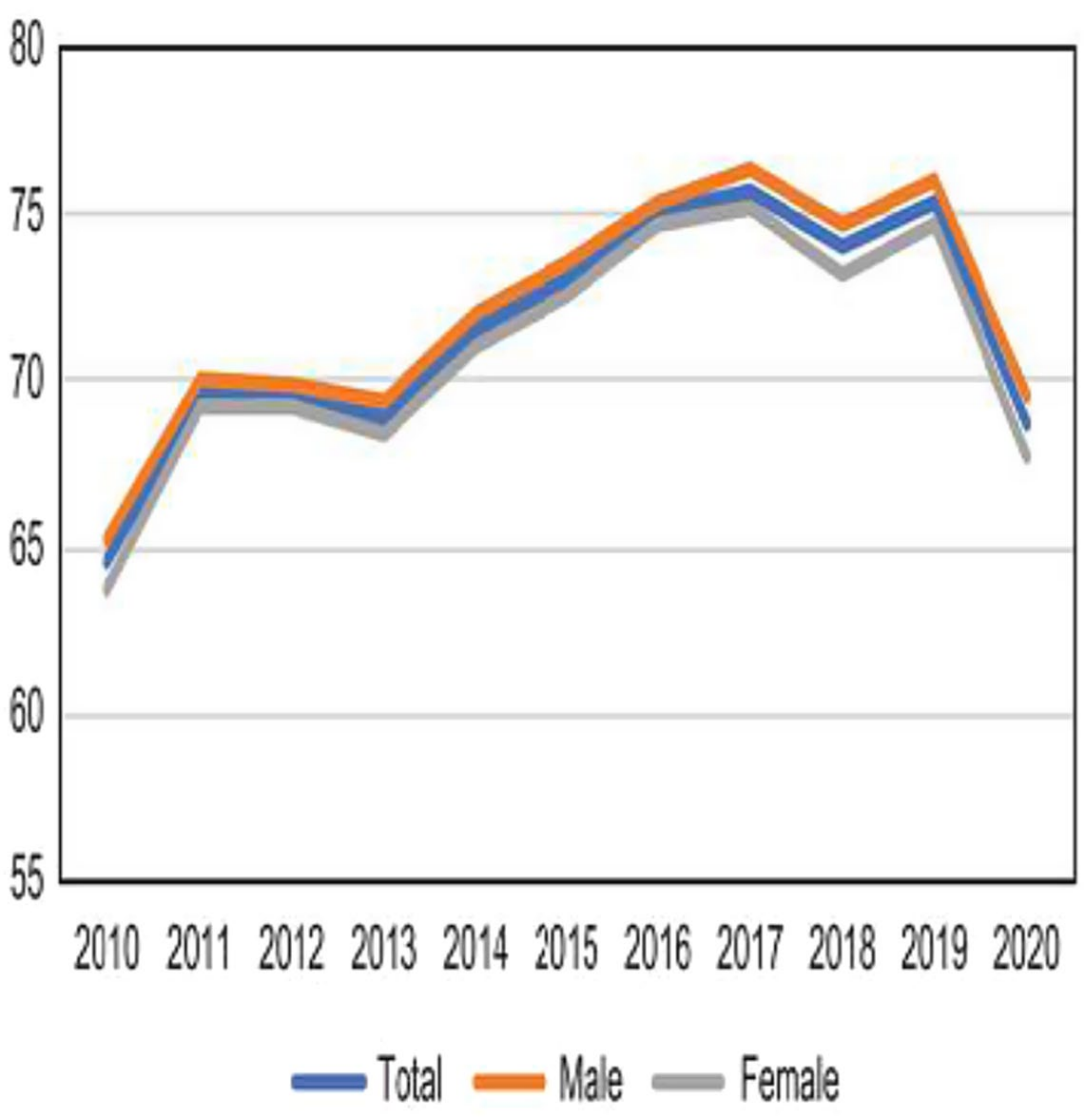
Trends in national general health screening participation, 2010–2020 announced by Statistics Korea.

Before the COVID-19 pandemic, because of proactive promotion of health checkups by the government and hospitals, the national health checkup rate had been steadily increasing. However, with the onset of the COVID-19 pandemic in 2020, the national health checkup rate plummeted (Figure 1).

According to mortality statistics reported by Statistics Korea, the leading causes of death remained largely consistent between 2019 and 2020, with cancer, heart disease, and pneumonia topping the list (4, 5). Despite the COVID-19 pandemic, the rankings and mortality rates of causes of death showed no significant difference, except for sepsis. In addition to mortality, these chronic diseases significantly challenge daily functioning and have a considerable economic impact (6).

Studies investigating the association between health checkups and chronic disease diagnoses have consistently demonstrated that national health examinations reduce the incidence and mortality rates of stroke and myocardial infarction (MI) (7, 8). Recent studies have also highlighted the challenges in post-pandemic screening recovery (9) and the importance of maintaining preventive services (10). Moreover, public health researchers support the claim that routine general healthcare screening can effectively reduce the incidence and mortality rates of chronic diseases (7, 10–14).

Therefore, whether during a pandemic or not, early detection and management of chronic diseases help reduce mortality rates. This study aims to answer the following research question: How did the pandemic-induced decline in screening rates influence the detection of major chronic diseases? We hypothesized that while screening participation decreased, alternative diagnostic pathways would mitigate the impact on disease detection. We analyzed the health checkup rate and number of newly diagnosed cases of chronic diseases in 2019, before the COVID-19 pandemic, and during the period corresponding to the COVID-19 pandemic in 2020.

## 2 Methods

### 2.1 National screening program in South Korea

South Korea operates a national health checkup program. The target population comprised adults aged ≥20 years, allowing for a health checkup every 2 years. The tests include medical examination, blood pressure measurement, fasting blood glucose testing, and lipid profile testing. The NHIS oversees the national health screening program and repeatedly encourages individuals who have not undergone screening to do so.

### 2.2 Study design and population

This study employed a retrospective cohort design that focused on adults aged ≥40 years. This study used data encompassing the national general checkup rates from January 2010 to December 2020 and the incidence rates of chronic diseases from January 2019 to December 2020. The primary objectives were to assess fluctuations in health examination rates and analyze the rates of new diagnoses of chronic diseases before and after the onset of the COVID-19 pandemic.

### 2.3 Data collection

Data from the Korea Disease Control and Prevention Agency-COVID-19-National Health Insurance Service (K-COV-N) were used. Chronic diseases were classified into categories including HTN, DM, DL, cardiovascular disease, cerebrovascular disease, and joint diseases. The diagnostic codes used were based on KCD-7 and ICD-10. Specific codes included HTN (I10–15), DM (E10–14), and DL (E78) (11).

To ensure the identification of new diagnoses, if the diagnostic code for a chronic disease was previously recorded at least once, it was excluded from the analysis. We included adults aged ≥40 years who had at least one NHIS record between 2019 and 2020. Missing or duplicate entries (<0.3%) were removed according to NHIS data-validation procedures.

Participants were categorized as “screened” if they completed a health checkup in the given year and “not screened” otherwise. Furthermore, the calculations included only individuals with examination records for a given year.

### 2.4 Ethical approval

All procedures in this study were conducted in compliance with relevant laws and institutional guidelines and were approved by the Institutional Review Board (IRB) of Kyung Hee University Hospital. The privacy rights of all human subjects were strictly protected, and data were handled in accordance with institutional and regulatory guidelines.

### 2.5 Statistical analysis

Using percentages, the proportion of individuals who underwent health checkups was determined. We calculated relative risks (RRs) and 95% confidence intervals (CIs) to quantify the difference in new diagnosis rates between the screened and non-screened groups. RRs were calculated using the formula RR = [a/(a + b)] / [c/(c + d)], and 95% CIs were derived using the Wald method. Analyses were stratified by sex and 10-year age groups to partially control for confounding factors.

All analyses were performed using SAS version 9.4 (SAS Institute, Cary, NC, USA). Two-tailed *p*-values < 0.05 were considered statistically significant. Due to the anonymized nature of the K-COV-N dataset, detailed socioeconomic status (SES) and regional variables were unavailable for analysis; this limitation is further addressed in the Discussion section.

## 3 Results

### 3.1 General health screening from 2010 to 2020

The number of health checkups increased from 2010 (7.7 million cases, 64.6%) to 2019 (11.6 million cases, 75.54%) but decreased in 2020 (11.4 million cases, 68.7%) during the COVID-19 pandemic. Except for 2018 and 2019, most examinees were in their 40s, and there were more male examinees than female examinees in all years (Figure 1 and Table 1).

**Table 1:** Numbers and ratios of individuals who underwent health checkups, 2010–2020 (*N*, %) according to year, age group, and sex.

Age (years)		2010, N (%)	2011, N (%)	2012, N (%)	2013, N (%)	2014, N (%)	2015, N (%)	2016, N (%)	2017, N (%)	2018, N (%)	2019, N (%)	2020, N (%)
Total	All	7,671,714 (64.59)	8,019,396 (69.65)	8,464,871 (69.59)	8,634,169 (68.87)	9,486,352 (71.52)	10,238,371 (73.1)	10,724,742 (75.02)	10,995,640 (75.75)	11,219,154 (73.96)	11,592,643 (75.39)	10,392,121 (68.72)
	40–49	2,893,186 (63.25)	2,987,807 (68.59)	3,038,660 (68.55)	3,139,333 (68.58)	3,338,836 (72)	3,562,858 (74.06)	3,640,093 (76.41)	3,646,628 (77.47)	3,530,415 (76.71)	3,633,855 (77.54)	3,124,468 (70.69)
	50–59	2,478,099 (67.33)	2,631,127 (72.47)	2,830,566 (71.87)	2,833,405 (70.35)	3,144,926 (72.76)	3,379,521 (73.81)	3,494,580 (75.92)	3,567,171 (76.71)	3,643,103 (75.57)	3,711,838 (76.44)	3,350,420 (70.77)
	60–69	1,470,560 (71.27)	1,482,665 (76.18)	1,594,604 (75.45)	1,620,317 (74.99)	1,836,196 (76.37)	2,050,946 (77.75)	2,238,568 (78.99)	2,348,001 (79.41)	2,485,591 (77.64)	2,638,830 (78.4)	2,512,142 (71.8)
	≥70	829,869 (53.25)	917,797 (58.08)	1,001,041 (59.59)	1,041,114 (58.81)	1,166,394 (61.38)	1,245,046 (62.94)	1,351,501 (64.51)	1,433,840 (65.15)	1,560,045 (61.3)	1,608,120 (65.11)	1,405,091 (56.88)
Male	All	3,948,541 (65.35)	4,079,129 (70.14)	4,328,723 (69.9)	4,380,086 (69.36)	4,840,238 (72.05)	5,227,472 (73.62)	5,435,966 (75.38)	5,550,927 (76.32)	5,684,963 (74.78)	5,840,617 (76.04)	5,307,281 (69.6)
	40–49	1,576,828 (63.86)	1,619,383 (69.15)	1,647,060 (69.07)	1,698,117 (69.57)	1,807,877 (72.89)	1,921,254 (74.96)	1,955,776 (77.22)	1,954,705 (78.49)	1,921,820 (78.01)	1,963,973 (78.96)	1,734,492 (72.65)
	50–59	1,269,410 (66.25)	1,316,175 (70.83)	1,452,881 (70.37)	1,423,442 (68.72)	1,612,856 (71.57)	1,730,669 (72.41)	1,771,386 (74.36)	1,791,118 (75.31)	1,837,779 (74.15)	1,856,608 (75.17)	1,690,115 (69.36)
	60–69	728,561 (71.01)	731,667 (75.66)	779,207 (74.4)	792,334 (73.89)	901,031 (75.27)	1,010,923 (76.75)	1,098,299 (77.67)	1,152,770 (78.24)	1,217,817 (76.15)	1,282,265 (76.94)	1,219,527 (70.37)
	≥70	373,742 (59.21)	411,904 (63.49)	449,575 (64.57)	466,193 (63.8)	518,474 (65.86)	564,626 (68.03)	610,505 (69.19)	652,334 (70.04)	707,547 (66.69)	737,771 (69.74)	663,147 (62.08)
Female	All	3,723,173 (63.81)	3,940,267 (69.16)	4,136,148 (69.27)	4,254,083 (68.38)	4,646,114 (70.98)	5,010,899 (72.57)	5,288,776 (74.65)	5,444,713 (75.19)	5,534,191 (73.13)	5,752,026 (74.74)	5,084,840 (67.82)
	40–49	1,316,358 (62.52)	1,368,424 (67.93)	1,391,600 (67.95)	1,441,216 (67.45)	1,530,959 (70.97)	1,641,604 (73.03)	1,684,317 (75.49)	1,691,923 (76.32)	1,608,595 (75.21)	1,669,882 (75.94)	1,389,976 (68.4)
	50–59	1,208,689 (68.5)	1,314,952 (74.2)	1,377,685 (73.52)	1,409,963 (72.07)	1,532,070 (74.07)	1,648,852 (75.33)	1,723,194 (77.59)	1,776,053 (78.18)	1,805,324 (77.07)	1,855,230 (77.75)	1,660,305 (72.26)
	60–69	741,999 (71.52)	750,998 (76.69)	815,397 (76.48)	827,983 (76.08)	935,165 (77.46)	1,040,023 (78.76)	1,140,269 (80.32)	1,195,231 (80.57)	1,267,774 (79.14)	1,356,565 (79.82)	1,292,615 (73.21)
	≥70	456,127 (49.18)	505,893 (54.32)	551,466 (56.07)	574,921 (55.31)	647,920 (58.22)	680,420 (59.26)	740,996 (61.11)	781,506 (61.57)	852,498 (57.44)	870,349 (61.64)	741,944 (52.92)

*N*: number of individuals who underwent health checkups.

%: (number of individuals who underwent health checkups divided by the number of individuals eligible for health checkups according to age group) × 100.

### 3.2 New diagnoses and changes in the diagnoses of chronic diseases

In 2020, new diagnoses of chronic diseases increased by 552,538 compared with the previous year (2019). As shown in Table 2, the diagnosis rates of HTN, DM, and DL increased by 0.49, 0.4, and 0.9%, respectively. Among cardiovascular diseases, the diagnosis rates of angina (−0.15%) and MI (−0.07%) decreased. Among cerebrovascular diseases, the diagnosis rates of CH (−0.02%) and cerebral infarction (−0.1%) decreased. Detailed stratifications by age and sex are provided in Supplementary Table S1.

**Table 2:** Prevalence (*N*, %) and changes in the diagnoses of chronic diseases between 2019 and 2020.

Diagnosis	Total			Male			Female		
	2019, N (%)	2020, N (%)	Change	2019, N (%)	2020, N (%)	Change	2019, N (%)	2020, N (%)	Change
Total	28,995,763 (100)	29,548,301 (100)	552,538 (0)	14,039,493 (100)	14,315,625 (100)	276,132 (0)	14,956,270 (100)	15,232,676 (100)	276,406 (0)
Hypertension	9,735,495 (33.58)	10,068,281 (34.07)	332,786 (0.49)	4,879,765 (34.76)	5,082,321 (35.5)	202,556 (0.74)	4,855,730 (32.47)	4,985,960 (32.73)	130,230 (0.26)
Diabetes mellitus	6,470,991 (22.32)	6,714,221 (22.72)	243,230 (0.4)	3,285,561 (23.4)	3,427,355 (23.94)	141,794 (0.54)	3,185,430 (21.3)	3,286,866 (21.58)	101,436 (0.28)
Dyslipidemia	10,806,197 (37.27)	11,279,735 (38.17)	473,538 (0.9)	5,045,544 (35.94)	5,294,398 (36.98)	248,854 (1.04)	5,760,653 (38.52)	5,985,337 (39.29)	224,684 (0.77)
Angina	1,562,856 (5.39)	1,549,149 (5.24)	−13,707 (−0.15)	848,406 (6.04)	855,393 (5.98)	6,987 (−0.06)	714,450 (4.78)	693,756 (4.55)	−20,694 (−0.23)
Myocardial infarction	730,146 (2.52)	725,233 (2.45)	−4,913 (−0.07)	445,954 (3.18)	453,322 (3.17)	7,368 (−0.01)	284,192 (1.9)	271,911 (1.79)	−12,281 (−0.11)
Heart failure	786,684 (2.71)	827,528 (2.8)	40,844 (0.09)	382,853 (2.73)	408,363 (2.85)	25,510 (0.12)	403,831 (2.7)	419,165 (2.75)	15,334 (0.05)
Cerebral hemorrhage	153,574 (0.53)	150,564 (0.51)	−3,010 (−0.02)	76,752 (0.55)	75,216 (0.53)	−1,536 (−0.02)	76,822 (0.51)	75,348 (0.49)	−1,474 (−0.02)
Cerebral infarction	859,352 (2.96)	845,262 (2.86)	−14,090 (−0.1)	443,109 (3.16)	442,445 (3.09)	−664 (−0.07)	416,243 (2.78)	402,817 (2.64)	−13,426 (−0.14)
Osteoarthritis	7,029,165 (24.24)	6,795,735 (23)	−233,430 (−1.24)	2,503,485 (17.83)	2,464,697 (17.22)	−38,788 (−0.61)	4,525,680 (30.26)	4,331,038 (28.43)	−194,642 (−1.83)
Osteoporosis	2,827,884 (9.75)	2,831,915 (9.58)	4,031 (−0.17)	310,827 (2.21)	314,046 (2.19)	3,219 (−0.02)	2,517,057 (16.83)	2,517,869 (16.53)	812 (−0.3)

*N*: number of new diagnoses of chronic diseases.

%: (number of individuals aged ≥ 40 years with new diagnoses of chronic diseases divided by the population aged > 40 years) × 100.

Change: difference between the 2019 and 2020 numbers.

### 3.3 Relative risks of new diagnoses among screened vs. non-screened individuals

Compared with the period before the COVID-19 pandemic, the relative risk (RR) for new diagnoses following the pandemic was >1 for HTN, DM, and DL among those who underwent screenings. As detailed in Table 3, the RRs (95% CI) were 1.0266 (1.0253–1.0278) for HTN, 1.0447 (1.0431–1.0464) for DM, and 1.0553 (1.0543–1.0564) for DL.

**Table 3:** Numbers of new chronic diagnoses (*N*) and percentages of patients who underwent health checkups between 2019 and 2020.

	Total			Male			Female		
	2019, N (%)	2020, N (%)	RR^a^	2019, N (%)	2020, N (%)	RR^a^	2019, N (%)	2020, N (%)	RR^a^
Hypertension	31,771	32,615	1.0266 (1.0253–1.0278)	34,262	35,345	1.0316 (1.03–1.0333)	29,242	29,766	1.0179 (1.016–1.0198)
Diabetes mellitus	21,480	22,440	1.0447 (1.0431–1.0464)	22,930	23,879	1.0414 (1.0392–1.0436)	20,007	20,938	1.0465 (1.0441–1.049)
Dyslipidemia	39,957	42,167	1.0553 (1.0543–1.0564)	38,767	40,874	1.0543 (1.0528–1.0559)	41,165	43,517	1.0571 (1.0557–1.0586)
Angina	4,775	4,661	0.9761 (0.9724–0.9798)	5,496	5,446	0.9907 (0.9859–0.9956)	4,043	3,843	0.9504 (0.9448–0.956)
Myocardial infarction	2,121	2079	0.9806 (0.975–0.9862)	2,753	2,741	0.9958 (0.9888–1.0027)	1,479	1,389	0.9392 (0.9299–0.9485)
Heart failure	2020	2085	1.0323 (1.0263–1.0383)	2,159	2,262	1.0476 (1.0394–1.0558)	1879	1901	1.0117 (1.0031–1.0205)
Cerebral hemorrhage	316	294	0.9287 (0.9147–0.9429)	319	294	0.9194 (0.9–0.9391)	314	294	0.9383 (0.9182–0.9589)
Cerebral infarction	1995	1865	0.9349 (0.9294–0.9405)	2,220	2,106	0.9488 (0.9413–0.9563)	1767	1,614	0.9134 (0.9051–0.9218)
Osteoarthritis	24,808	24,040	0.969 (0.9676–0.9704)	18,504	18,167	0.9818 (0.9793–0.9842)	31,209	30,169	0.9667 (0.9649–0.9684)
Osteoporosis	9,746	9,873	1.013 (1.0104–1.0156)	1985	2003	1.0095 (1.0012–1.0179)	17,627	18,086	1.0261 (1.0234–1.0287)

*N*: Cases per 100,000 individuals.

a RR: 2020/2019 numbers.

Table shows the differences in the numbers of new chronic disease diagnoses among individuals who underwent medical examinations during the 2 years before and after the onset of the COVID-19 pandemic (January 2019 to December 2020), using the relative risk (RR) as a measure.

Individuals who underwent a medical examination among the eligible population for screening (excluding those with examination records who were not part of the eligible population).

Conversely, cardiovascular diseases showed RRs of 0.9761 (95% CI, 0.9724–0.9798) for angina and 0.9806 (95% CI, 0.975–0.9862) for MI. Among cerebrovascular diseases, the RRs were 0.9287 (95% CI, 0.9147–0.9429) for CH and 0.9349 (95% CI, 0.9294–0.9405) for cerebral infarction.

## 4 Discussion

This study identified the association between health checkups and new diagnoses of chronic diseases. Whether the COVID-19 pandemic increased or decreased the prevalence of chronic diseases remains controversial. Several studies have reported conflicting findings (15).

However, before and after the COVID-19 pandemic, the number of new diagnoses of chronic diseases, such as HTN, DM, and DL, increased. Notably, the RR of a new chronic disease diagnosis for the 2 years before and after COVID-19 was consistently higher among those who underwent health checkups than among those who did not (16).

In Korea, the steadily increasing health checkup rates from 2010 to 2019 can be attributed to the concerted efforts of the government. However, the impact of the COVID-19 pandemic has been significant and rapidly decreased the number of individuals undergoing health checkups. This decline can be attributed to reduced hospital visits and postponement of scheduled examinations because of the pandemic (16). Behavioral barriers, including fear of infection and reduced willingness to participate in preventive health screenings, have also been documented during the pandemic (19–21).

Despite the decrease in health screening rates, we observed more newly diagnosed chronic diseases. This finding suggests a behavioral and systemic shift rather than a direct causal contradiction. The expansion of telemedicine and pharmacy-based monitoring partially compensated for reduced screening access, allowing for the continued identification of symptomatic cases. Although the overall hospital utilization rate decreased, awareness of individual health increased, which may have increased new diagnoses of certain chronic diseases through alternative diagnostic pathways.

Comparing internationally, South Korea’s rapid resumption of preventive services contrasts with countries like the United Kingdom, where screening backlogs persisted longer. Similar to Japan, Korea’s universal health insurance system provided a resilient foundation, although disparities remained.

The numbers of new diagnoses of cardiovascular diseases other than HF, particularly angina, MI, and cerebrovascular disease, were similar before and after the COVID-19 pandemic. These diseases are often diagnosed after emergency room treatment rather than routine examinations. Previous studies have reported reduced emergency department visits and decreased in-person outpatient care utilization during the COVID-19 pandemic (16–18). Thus, their new diagnosis may not have been closely related to a checkup or clinic visit.

### 4.1 Limitations

This study has several limitations. First, the observation period was limited to 2 years (2019–2020), which may not capture the long-term effects of the pandemic. Second, due to the nature of the K-COV-N database, we could not control for detailed socioeconomic status or regional variations (urban vs. rural), which are known confounders in healthcare access. Third, there is a potential selection bias, as individuals who are more health-conscious may have been more likely to undergo screening during the pandemic, potentially skewing the detection rates. Finally, our findings represent associations and should be interpreted with caution regarding causality.

## 5 Conclusion

The COVID-19 pandemic disrupted preventive healthcare but did not eliminate chronic disease detection in South Korea. The findings suggest that while population-level screening decreased, healthcare systems and individuals adapted through alternative diagnostic routes.

To strengthen health system resilience against future public health emergencies, we recommend integrating digital health strategies and ensuring equity-oriented screening programs. As noted by recent studies, the digital transformation of preventive services is crucial for maintaining continuity of care. These findings underscore the importance of resilient yet uneven health systems that maintain continuity of essential services during public health crises.

## Data Availability

The data analyzed in this study is subject to the following licenses/restrictions: The data supporting the findings of this study are available from the corresponding author upon reasonable request. Requests to access these datasets should be directed to Bumjo Oh, bo39@snu.ac.kr.
