# Supplementary material for: Impact of COVID-19 on national health screening participation and chronic disease detection: evidence from South Korea’s NHIS database
Source: Front Public Health. 2026 Mar 18;13:1721010. doi: 10.3389/fpubh.2025.1721010 (PMC13039092; doi:10.3389/fpubh.2025.1721010)
Supplement: Supplementary file 1 [file Table_1.docx]

**Supplementary Material**

**Supplementary Table S1.** Numbers of new chronic disease diagnoses (N) and changes in 2019 and 2020 among individuals who did not undergo medical examinations.

|  | Total | | | Male | | | Female | | |
| --- | --- | --- | --- | --- | --- | --- | --- | --- | --- |
|  | 2019 | 2020 | RR* | 2019 | 2020 | RR* | 2019 | 2020 | RR* |
| Hypertension | 33217 | 33372 | 1.0047  (1.0027–1.0066) | 32736 | 33220 | 1.0148  (1.012–1.0176) | 33672 | 33517 | 0.9954  (0.9928–0.998) |
| Diabetes mellitus | 21468 | 21421 | 0.9978  (0.9952–1.0004) | 22322 | 22405 | 1.0037  (1.0001–1.0073) | 20659 | 20475 | 0.9911  (0.9874–0.9948) |
| Dyslipidemia | 31657 | 32535 | 1.0277  (1.0257–1.0298) | 30621 | 31637 | 1.0332  (1.0302–1.0362) | 32639 | 33398 | 1.0232  (1.0205–1.026) |
| Angina | 5464 | 5250 | 0.9608  (0.9554–0.9663) | 6007 | 5848 | 0.9735  (0.9661–0.9811) | 4949 | 4675 | 0.9445  (0.9366–0.9525) |
| Myocardial infarction | 2775 | 2630 | 0.9476  (0.94–0.9553) | 3428 | 3349 | 0.9771  (0.967–0.9872) | 2158 | 1939 | 0.8986  (0.887–0.9104) |
| Heart failure | 3150 | 3067 | 0.9736  (0.9663–0.981) | 3023 | 3021 | 0.9993  (0.9884–1.0104) | 3270 | 3111 | 0.9513  (0.9415–0.9612) |
| Cerebral hemorrhage | 736 | 650 | 0.8827  (0.8685–0.8971) | 772 | 678 | 0.8777  (0.8581–0.8977) | 702 | 623 | 0.8874  (0.8671–0.9081) |
| Cerebral infarction | 3723 | 3375 | 0.9067  (0.9004–0.9131) | 3845 | 3531 | 0.9182  (0.9092–0.9273) | 3607 | 3226 | 0.8946  (0.8856–0.9036) |
| Osteoarthritis | 20587 | 19786 | 0.9611  (0.9585–0.9637) | 14741 | 14448 | 0.9801  (0.9755–0.9847) | 26122 | 24915 | 0.9538  (0.9507–0.9569) |
| Osteoporosis | 7818 | 7806 | 0.9985  (0.9938–1.0031) | 1999 | 1943 | 0.9721  (0.9589–0.9855) | 13327 | 13438 | 1.0084  (1.0035–1.0132) |

N: Cases per 100,000 individuals.

*RR: 2020/2019 numbers

Table shows the differences in the numbers of new chronic disease diagnoses among individuals who did not undergo medical examinations 2 years before and after the onset of the COVID-19 pandemic, using the relative risk (RR) as a measure.
